# Gene Expression Profiling in Huntington’s Disease: Does Comorbidity with Depressive Symptoms Matter?

**DOI:** 10.3390/ijms21228474

**Published:** 2020-11-11

**Authors:** Gabriela Delevati Colpo, Natalia Pessoa Rocha, Erin Furr Stimming, Antonio Lucio Teixeira

**Affiliations:** 1 Neuropsychiatry Program, Louis A Faillace Department of Psychiatry and Behavioral Sciences, McGovern Medical School, The University of Texas Health Science Center at Houston, Houston, TX 77054, USA; Gabriela.D.Colpo@uth.tmc.edu; 2 HDSA Center of Excellence at The University of Texas Health Science Center at Houston, Houston, TX 77030, USA; Natalia.PessoaRocha@uth.tmc.edu (N.P.R.); Erin.E.Furr@uth.tmc.edu (E.F.S.); 3 Department of Neurology, McGovern Medical School, The University of Texas Health Science Center at Houston, Houston, TX 77030, USA; 4 Instituto de Ensino & Pesquisa, Santa Casa BH, Belo Horizonte 30150-221, Brazil

**Keywords:** Huntington’s disease, neurodegenerative disease, RNA-seq, depression, neurodevelopment

## Abstract

Huntington’s disease (HD) is an inherited neurodegenerative disease. Besides the well-characterized motor symptoms, HD is marked by cognitive impairment and behavioral changes. In this study, we analyzed the blood of HD gene carries using RNA-sequencing techniques. We evaluated samples from HD gene carriers with (*n* = 8) and without clinically meaningful depressive symptoms (*n* = 8) compared with healthy controls (*n* = 8). Groups were age- and sex-matched. Preprocessing of data and between-group comparisons were calculated using DESeq2. The Wald test was used to generate *p*-values and log2 fold changes. We found 60 genes differently expressed in HD and healthy controls, of which 21 were upregulated and 39 downregulated. Within HD group, nineteen genes were differently expressed between patients with and without depression, being 6 upregulated and 13 downregulated. Several of the top differentially expressed genes are involved in nervous system development. Although preliminary, our findings corroborate the emerging view that in addition to neurodegenerative mechanisms, HD has a neurodevelopmental component. Importantly, the emergence of depression in HD might be related to these mechanisms.

## 1. Introduction

Huntington’s disease (HD) is an inherited autosomal dominant neurodegenerative disease. HD is caused by expanded CAG trinucleotide repeats in the exon 1 of the *Huntingtin* gene (*HTT*), which encodes a mutant form of the huntingtin protein (HTT) with an abnormal polyglutamine tail at the N-terminus [1,2,3]. HTT is an important protein for neurodevelopment. The knockout of the *HTT* in mice results in major brain abnormalities and death soon after birth [4,5,6]. In addition, depletion of wild-type HTT in the postnatal mouse brain leads to progressive neurodegeneration [7]. The presence of 40 or more CAG repeats invariably causes the disease, and longer repeats predict earlier disease onset. Mutant HTT (mHTT) is widely expressed and believed to induce neurodegeneration through different mechanisms, including abnormal interaction with other proteins, leading to cellular changes and ultimately cell death [8]. 

HD is marked by a triad of symptoms including motor, cognitive and psychiatric or behavioral symptoms. The clinical diagnosis of HD has been historically based on motor symptoms. However, cognitive and psychiatric symptoms are often present years prior to the onset of clinically relevant motor symptoms [9]. Psychiatric symptoms, such as anxiety, irritability, impulsivity, and depressed mood are frequent in individuals with HD and can be quite troublesome. Major depression is the most common psychiatric syndrome among premanifest HD carriers [10]. Depression may precede the onset of typical motor symptoms by 4 to 10 years, making it one of the earliest signs possibly related to HD pathophysiology. Moreover, around 40–50% of patients with HD experience depression at some point during the course of the disease [11], and many patients and their relatives consider this problem the most distressing aspect of the illness. Actually, depression seems to influence more profoundly health-related quality of life in HD than motor symptoms or cognitive dysfunction [12]. The presence of depression in HD is also an important predictor of suicidal behavior [13]. Patients with HD have been shown to commit suicide four to eight times more often than the general population [14,15,16]. The increased rate of suicide may be related to several factors, including the emotional distress of having an incurable disease alongside the elevated frequency of depression and other behavioral symptoms, such as impulsivity [13,17]. 

The reasons for the frequent co-occurrence of HD and depression are still unclear. The heritability nature of HD does not fully explain the high prevalence of psychiatric symptoms, especially depression, in HD [18,19]. Importantly, as the severity of depression is not associated with disease progression [20,21,22], different mechanisms are likely to affect neurons involved in mood regulation circuits and neurons involved in motor skills that are impaired in manifest or later stages of the disease. The molecular underpinnings of psychiatric symptoms in HD are poorly understood. Therefore, we designed an exploratory study to investigate peripheral blood gene expression profile through RNA-seq in HD gene carriers (with and without depression) and healthy controls. Our hypothesis is that HD gene carriers presenting with depression have a different transcriptome profile when compared with HD gene carriers without these symptoms. In addition, we assessed different genetic pathways underlying HD in comparison with healthy controls. This is the first study to evaluate gene expression profile comparing these subgroups of HD gene carriers and healthy controls. Our results advance the understanding of the biological mechanisms associated with depression in HD with the ultimate goal of identifying a more specific and effective anti-depressant strategy to improve the quality of life and decrease suicide rates of these subjects [23].

## 2. Results

Demographic data from HD gene carriers and controls are shown in Table 1. Groups did not differ in age, years of education, sex and BMI. 

### 2.1. Differential Gene Expression Analysis

Sixty genes were differently expressed in HD gene carriers (*n* = 16) compared with healthy controls (*n* = 8), of which 21 were upregulated and 39 were downregulated (Table 2). When analyzing HD gene carriers with depression versus non-depression, there were 19 genes differently expressed, being 6 upregulated and 13 downregulated (Table 3). 

### 2.2. Gene Ontology Analysis

Comparing HD gene carriers and healthy controls, 109 enriched pathways were identified (Table 4). Among the enriched pathways, many of them contain the top differentially expressed gene *ADGRG1*, such as GO:0010573~vascular endothelial growth factor production, GO:0021801~cerebral cortex radial glia guided migration, GO:0021796~cerebral cortex regionalization GO:0021819~layer formation in cerebral cortex. Noteworthy, there were other enriched pathways with different genes involved in neurodevelopment, such as the GO:2001224~positive regulation of neuron migration and GO:0007155~cell adhesion. 

In the comparison among HD gene carriers, 61 enriched pathways were identified (Table 5). Several pathways have the *NECTIN2* gene, as GO:0002891~positive regulation of immunoglobulin mediated immune response, GO:0034332~adherens junction organization, GO:0007157~heterophilic cell-cell adhesion via plasma membrane cell adhesion molecules. 

### 2.3. Validation of Microarray Data by Real-Time qPCR

**Figure 1:**
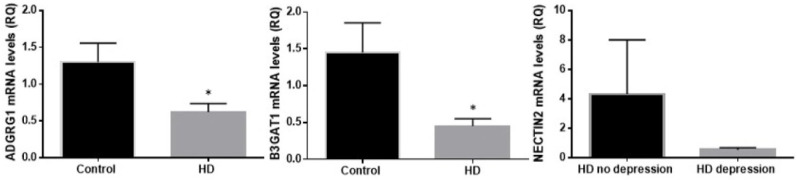
mRNA levels of the top three differentially expressed genes. *: *p* < 0.05.

We validated the RNA-seq results by determining mRNA levels of the top three differentially expressed genes (Figure 1). Regarding *ADGRG1* and *B3GAT1*, real-time qPCR confirmed RNAseq results. For *NECTIN2*, the results showed the same tendency, but without reaching statistical significance. 

## 3. Discussion

In this study, we examined RNA-seq gene expression in the blood of HD gene carriers with and without depressive symptoms and healthy controls. Differences between HD gene carriers and controls were marked, with several genes related to neurodevelopmental pathways. Conversely, differences among HD gene carriers with and without depression were less pronounced. As several of the genes and enriched pathways are involved in the development of the nervous system, our preliminary findings corroborate the emerging view that in addition to being a neurodegenerative disease, HD has a neurodevelopmental component. 

The gene *B3GAT1* was among the top differentially expressed genes between HD gene carriers and controls. This gene is the key enzyme during the biosynthesis of the carbohydrate epitope HNK-1. The HNK-1 epitope is mainly present in the brain, more specifically in a number of cell adhesion molecules (CAM) important for neuronal cell adhesion, synaptic plasticity, and, therefore, neurodevelopment [24,25]. *B3GAT1* has also been implicated in major psychiatric disorders, as schizophrenia and schizoaffective disorders [26]. Mice deficient for *B3GAT1* exhibited normal development of gross anatomical features, but had impairments of high-order brain functions, including learning, and memory [25].

Another gene differentially expressed between HD gene carriers and healthy controls was *ADGRG1* (or *GPR56*). This gene encodes a member of the G protein-coupled receptor family. ADGRG1 plays critical roles in the development of several organs, including the brain, with extensive implications for human diseases and their treatment [27,28,29,30,31]. Mutations in *ADGRG1* cause a severe human brain malformation called bilateral frontoparietal polymicrogyria, characterized by cortical lamination defects, cerebellar hypoplasia, and central nervous system hypomyelination [32,33]. In addition, *ADGRG1* regulates embryonic brain development and postnatal myelination [34,35]. 

Both *B3GAT1* and *ADGRG1* are involved in neurodevelopment, corroborating recent studies that propose the conceptualization of HD as a neurodevelopmental disease [36,37]. It has been proposed that CAG repeat expansion may be phylogenetically relevant for brain development [38]. Animal models, in vitro studies and molecular research studies have shown that the protein HTT mediates a variety of developmental processes in the central nervous system [36,39]. Accordingly, the presence of mHTT may influence neuronal homeostasis throughout development, ultimately leading to premature cell death and, hence, neurodegeneration from otherwise nonlethal stressors. Importantly, prior to neuronal death, mHTT may cause subclinical neurodevelopmental abnormalities [40,41]. In humans, a study evaluating fractal dimension (FD), a sensitive measure of cortical neurodevelopment, showed that the premanisfest HD subjects differed from healthy controls in the amount of cortical folding in temporal regions, and in motor and visual areas. This spatial pattern of FD differs from what has been observed in well-defined neurodevelopmental disorders such as autism. In this latter case, higher cortical folding was seen in frontal, temporal and parietal regions, and more pronounced in children than in young adults [42]. Altogether these findings suggest that *HTT* gene expression may be a factor that contributes to cortical development, especially those regions that differ between patients and controls [43]. 

Comparing HD gene carriers with depression and without depression, one of the top differentially expressed gene was *NECTIN2*. *NECTIN2* is downregulated in HD gene carriers with depression. The correspondent protein is a CAM, i.e., an important structural substrate required for synaptic plasticity and synaptogenesis. NECTIN2 is part of the nectin family of four structurally similar type-I membrane glycoproteins belonging to the immunoglobulin superfamily (IgSF) [44]. CAMs are trans-synaptic anchors and mediators of experience-dependent signaling, dynamically modulating synaptic activity and plasticity [45,46]. These proteins participate in synaptogenesis, neural growth, synaptic maturation, and modulate synaptic function through interactions with other synaptic proteins and receptors [45,47]. Nectins are also able to interact and activate membrane receptors of different growth factors, such as fibroblast growth factor, platelet-derived growth factor and the vascular endothelial growth factor, regulating proliferation, differentiation, and cell survival [48]. These interactions are relevant for depression given the role played by growth factors in its pathophysiology [49]. 

CAM dysfunction has been associated with several neuropsychiatric conditions [50,51,52,53]. In addition, chronic stress can affect the expression of CAMs, with the chronic restraint model of depression leading to decreased expression of CAMs in the hippocampus [52]. This observation goes in line with the concept that neurodevelopmental impairment and/or dysfunction increase the vulnerability to psychiatric disorders by altering the developmental programming of brain regions that are associated with affective and cognitive processing [54,55,56]. Indeed, the presence of neurodevelopmental abnormalities, notably involving cortical regions, is associated with an increased lifetime risk for depression [54]. The establishment of cortical thickness depends on different processes such as cell death, synaptogenesis, synaptic pruning, and myelination during the first two decades of life, with a dynamic synaptic reorganization modulated by environmental influences [57,58,59,60]. Abnormal brain volume has been consistently shown in depression, and a recent meta-analysis including over 10,000 subjects showed cortical abnormalities in adults and adolescents with major depression [61]. 

We also performed gene ontology (GO) analysis to identify the pathways that are enriched by differently expressed genes. The pathway ‘cerebral cortex radial glia guided migration’ has the presence of *ADGRG1*. This pathway is involved in neuronal radial migration in the developing cerebral cortex. The cerebral cortex has a well-organized six-layered architecture. The establishment of cortical layers in the mammal developing cortex requires an elaborate control of multiple processes, such as cell proliferation, differentiation, apoptosis, and neuronal migration [62]. Studies have revealed that radial glia are present during corticogenesis and their processes span the full thickness of the cortical wall [63]. Defects in radial glial cells could lead to cortical heterotopias, suggesting that normal radial glial cells are critical for cerebral cortex development [64,65]. One of the pathways containing *NECTIN2* is called ‘cell part morphogenesis’ that refers to how structures of a cell are generated and organized. Altogether, these results implicate the involvement of neurodevelopmental pathways in HD and, more specifically, in the emergence of depression. 

Interestingly, another enriched pathway in the comparison among HD carriers was the positive regulation of ‘immunoglobulin mediated immune response’ in subjects with depression. This pathway has *NECTIN2* and is involved in processes that activate or increase the frequency, rate, or extent of an immunoglobulin-mediated immune response. The immune system is a relevant player in the pathological cascade of neurodegenerative diseases triggered by misfolded proteins, including HD [66]. In addition, immune changes have been associated with the pathophysiology of depression [67]. 

There are limitations in this study that must be considered. First, this is a cross-sectional study with a small sample size. The analyses were performed in the blood and might not accurately reflect alterations in the central nervous system. Despite these shortcomings, this is the first study to compare peripheral (i.e., blood) gene expression profile between HD gene carriers and healthy controls. 

In summary, we found distinctive gene expression related to neurodevelopmental pathways in HD. These findings are in line with recent studies suggesting that HD is not only a neurodegenerative disease but also has a neurodevelopmental component. Our findings also suggest that this neurodevelopmental component can contribute to the increased rates of depression in HD. Future directions include analyses of the identified genes in larger cohorts of HD carriers aiming to better understand the pathophysiology and risk factors associated with depression (and other behavioral correlates) in HD. 

## 4. Material and Methods

### 4.1. Subjects and Clinical Assessments 

This study included 16 patients with a genetic diagnosis of HD (9 premanifest and 7 manifest HD, i.e., patients with a clinical diagnosis of HD), being eight HD gene carriers with symptoms of depression and eight patients without, and a group of 8 healthy controls. HD gene carriers were recruited from the Huntington Disease Society of America (HDSA) Center of Excellence at University of Texas Health Science Center at Houston (UTHealth). Controls were recruited from the local community, comprising a group of people with no history of neurological or psychiatric disorders. Genetic diagnosis was confirmed by a genotype CAG allele ≥ 36. A movement disorders specialist evaluated all patients and the clinical diagnosis of HD was based on established motor signs, i.e., a Diagnostic Confidence Level (DCL) set at 4 in the Unified HD Rating Scale (UHDRS) (1996). All subjects provided written informed consent before admission to the study (Approval Number: HSC-MS-17-0234, and issue on 10 May 2017). The Research Ethics Committees of UTHealth approved this study.

The clinical evaluation included a questionnaire about socio-demographic information alongside motor, cognitive and behavioral assessments. HD gene carriers were subjected to motor function evaluation with the UHDRS (1996). Behavioral symptoms were evaluated through the short version of the Problem Behaviors Assessment (PBS-s). The PBA-s is a semi-structured interview containing 11 items, each designed to measure the severity and frequency of different behavioral symptoms in HD (McNally G et al., 2015). Patients with a score zero or < 2 were considered without depression symptoms. Patients had moderate to severe symptoms of depression. 

### 4.2. Blood Sampling

Peripheral blood was collected from participants by venipuncture into PAXgene Blood RNA Tubes (PreAnalytix, QIAGEN, Inc., Germantown, MD, USA) on the same day of clinical assessment. RNA was isolated with the PreAnalytix kit (QIAGEN, Inc., Germantown, MD, USA) according to the manufacturer’s instructions. Total RNA samples were quantified and sent to a core facility to perform RNA-sequencing.

### 4.3. Gene Expression Analysis

RNA samples were quantified upon receipt using Qubit 2.0 Fluorometer (Life Technologies, Carlsbad, CA, USA) and RNA integrity was checked with 4200 TapeStation (Agilent Technologies, Palo Alto, CA, USA). rRNA depletion along with globin depletion was performed using Globin Zero Gold kit (Illumina, San Diego, CA, USA). 

RNA sequencing library preparation used NEBNext Ultra RNA Library Prep Kit for Illumina by following the manufacturer’s recommendations (NEB, Ipswich, MA, USA). Briefly, enriched RNAs were fragmented for 15 minutes at 94 °C. First strand and second strand cDNA were subsequently synthesized. cDNA fragments were end repaired and adenylated at 3’ends, and universal adapter was ligated to cDNA fragments, followed by index addition and library enrichment with limited cycle PCR. Sequencing libraries were validated using the Agilent Tapestation 4200 (Agilent Technologies, Palo Alto, CA, USA), and quantified by using Qubit 2.0 Fluorometer (Invitrogen, Carlsbad, CA, USA) as well as by quantitative PCR (Applied Biosystems, Carlsbad, CA, USA).

The sequencing libraries were multiplexed and clustered on two lanes of a flow cell and loaded on the Illumina HiSeq instrument according to manufacturer’s instructions. The samples were sequenced using a 2 × 150 Paired End (PE) configuration. Image analysis and base calling were conducted by the HiSeq Control Software (HCS). Raw sequence data (.bcl files) generated from Illumina HiSeq were converted into fastq files and de-multiplexed using Illumina’s bcl2fastq 2.17 software. One mismatch was allowed for index sequence identification.

### 4.4. Data Analysis

After demultiplexing, sequence data were checked for overall quality and yield. Then, sequence reads were trimmed to remove possible adapter sequences and nucleotides with poor quality using Trimmomatic v.0.36. The trimmed reads were mapped to the Homo sapiens reference genome GRCh38 available on ENSEMBL using the STAR aligner v.2.5.2b. The STAR aligner is a splice aware aligner that detects splice junctions and incorporates them to help align the entire read sequences. BAM files were generated as a result of this step. Unique gene hit counts were calculated by using feature Counts from the Subread package v.1.5.2. Only unique reads within exon regions were counted.

After extraction of gene hit counts, the gene hit counts table was used for downstream differential expression analysis. Using DESeq2, a comparison of gene expression between the groups of samples was performed. The Wald test was used to generate *p*-values and Log2 fold changes. Genes with adjusted *p*-values < 0.05 and absolute log2 fold changes > 1 were called as differentially expressed genes for each comparison. A gene ontology analysis was performed on the statistically significant set of genes by implementing the software GeneSCF v1.1. The GO list was used to cluster the set of genes based on their biological process and determine their statistical significance. A PCA analysis was performed using the "plotPCA" function within the DESeq2 R package. The plot shows the samples in a 2D plane spanned by their first two principal components. The top 500 genes, selected by highest row variance, were used to generate the plot.

### 4.5. Gene Ontology Analysis

A gene ontology analysis was performed on the statistically significant set of genes by implementing the software GeneSCF v.1.1-p2. The goa_human GO list was used to cluster the set of genes based on their biological processes and determine their statistical significance. A list of genes clustered based on their gene ontologies was generated, adjusted *p*-value less than 0.05 in the differentially expressed gene sets.

### 4.6. Real-Time Quantitative PCR

Three top differentially expressed genes between groups were selected for validation using real time quantitative PCR. Briefly, RNA samples (300 ng) were initially converted into cDNA using the High Capacity cDNA Synthesis Kit (Life Technologies, Carlsbad, CA) and later diluted 2 times for the PCR reactions. Amplifications of *Adhesion G Protein-Coupled Receptor G1* (*ADGRG1*), *Galactosylgalactosylxylosylprotein 3-beta-glucuronosyltransferase 1* (*B3GAT1*) and *Nectin Cell Adhesion Molecule 2* (*NECTIN2*) were performed in 12 μL-reactions using inventoried FAM-MGB-labeled TaqMan Gene Expression Assays (Hs00938474_m1, Hs01024500_m1, Hs01071562_m1 for *ADGRG1*, *B3GAT1* and *NECTIN2*, respectively) and the VIC-MGB_PL-labelled *beta-2-microglobulin* (*B2M*) as endogenous control (Hs00187842_m1). PCR reactions were run on a QuantStudio 7 Flex Real-Time PCR System (Life Technologies, Massachusetts, USA) with each sample assayed in triplicate. Data were analyzed by the 2(-Delta Delta C(T)) method. 

### 4.7. Statistical Analysis

After extraction of gene hit counts, the gene hit counts table was used for downstream differential expression analysis. Preprocessing of data and between-group comparisons were calculated using DESeq2. The Wald test was used to generate *p*-values and log2 fold changes. Genes with a *p*-value < 0.05 and absolute log2 fold change > 1 were called as differentially expressed genes. In addition, we clustered differentially expressed genes by their gene ontology (GO) using GeneSCF and the enrichment of GO terms was tested by Fisher exact test. In the PCR results we used t-tests with a significance level of *p* < 0.05 were used to evaluate for possible group differences.

## Figures and Tables

**Table 1:** Demographic variables of the subjects enrolled in this study.

Variables	Control Group (*n* = 8)	HD without Depression (*n* = 8)	HD with Depression (*n* = 8)	Statistic
Female sex	5 (62.5%)	5 (62.5%)	5 (62.5%)	χ2(1) = 0 *p* = 1
Age	51.91 (10.59)	38.17 (8.44)	48.27 (13.81)	F (2, 21) = 3.12 *p* = 0.067
Years of education	15.93 (4.82)	15.57 (3.59)	14.25 (2.96)	F (2, 21) = 0.4078 *p* = 0.6703
BMI	30.86 (8.09)	29.04 (7.15)	29.39 (6.18)	F (2, 20) = 0.3627 *p* = 0.7003

**Table 2:** Different expressed genes between HD gene carrier and healthy controls.

Gene ID	Gene Name	log2FoldChange	Stat	*p* Value	padj
ENSG00000109956	B3GAT1	1.484	4.332	0.000	0.019415
ENSG00000205336	ADGRG1	1.293	4.605	0.000	0.019415
ENSG00000165568	AKR1E2	−1.007	−4.185	0.000	0.026784
ENSG00000206172	HBA1	−1.208	−3.890	0.000	0.038152
ENSG00000110203	FOLR3	−1.687	−3.265	0.001	0.059707
ENSG00000234389	AC007278.1	−1.262	−3.162	0.002	0.068408
ENSG00000137267	TUBB2A	−1.591	−2.213	0.027	0.192338
ENSG00000196565	HBG2	−1.006	−2.166	0.030	0.201445
ENSG00000239839	DEFA3	1.101	2.017	0.044	0.234289
ENSG00000237973	MTCO1P12	2.565	3.763	0.000	NA
ENSG00000248527	MTATP6P1	1.036	3.042	0.002	NA
ENSG00000162631	NTNG1	−1.102	−2.618	0.009	NA
ENSG00000196539	OR2T3	−1.329	−3.064	0.002	NA
ENSG00000200488	RN7SKP203	−1.059	−2.842	0.004	NA
ENSG00000071909	MYO3B	1.142	2.789	0.005	NA
ENSG00000144485	HES6	−1.141	−3.027	0.002	NA
ENSG00000144908	ALDH1L1	−1.288	−2.189	0.029	NA
ENSG00000145362	ANK2	−1.017	−1.961	0.050	NA
ENSG00000178636	AC092656.1	−1.233	−3.359	0.001	NA
ENSG00000247199	AC091948.1	−1.128	−4.107	0.000	NA
ENSG00000230202	AL450405.1	2.670	4.467	0.000	NA
ENSG00000260997	AC004847.1	1.032	3.541	0.000	NA
ENSG00000075213	SEMA3A	−1.025	−2.605	0.009	NA
ENSG00000279483	AC090498.1	−1.257	−2.763	0.006	NA
ENSG00000276819	TRBV15	−1.360	−3.558	0.000	NA
ENSG00000201098	RNY1	−1.164	−2.702	0.007	NA
ENSG00000234449	FAM239A	−2.310	−2.901	0.004	NA
ENSG00000215374	FAM66B	−1.026	−4.054	0.000	NA
ENSG00000240905	RN7SL798P	1.118	3.315	0.001	NA
ENSG00000184350	MRGPRE	−1.929	−2.127	0.033	NA
ENSG00000006071	ABCC8	−1.368	−2.151	0.031	NA
ENSG00000170959	DCDC1	−1.127	−2.440	0.015	NA
ENSG00000156113	KCNMA1	−1.153	−2.707	0.007	NA
ENSG00000235602	POU5F1P3	1.022	3.920	0.000	NA
ENSG00000225231	LINC02470	−1.765	−2.060	0.039	NA
ENSG00000177359	AC024940.2	−1.687	−3.254	0.001	NA
ENSG00000273824	AC008033.3	1.148	2.862	0.004	NA
ENSG00000123201	GUCY1B2	−1.532	−3.138	0.002	NA
ENSG00000102837	OLFM4	1.081	2.248	0.025	NA
ENSG00000139926	FRMD6	−1.146	−3.784	0.000	NA
ENSG00000021645	NRXN3	−1.020	−2.433	0.015	NA
ENSG00000189419	SPATA41	−1.037	−3.232	0.001	NA
ENSG00000205918	PDPK2P	1.111	3.006	0.003	NA
ENSG00000261245	AC093520.2	1.096	2.797	0.005	NA
ENSG00000270124	AC092127.2	1.040	3.505	0.000	NA
ENSG00000262074	SNORD3B-2	−1.154	−2.194	0.028	NA
ENSG00000276241	AC243829.2	1.579	3.073	0.002	NA
ENSG00000274512	TBC1D3L	1.021	2.304	0.021	NA
ENSG00000142449	FBN3	−1.148	−1.982	0.047	NA
ENSG00000187244	BCAM	−1.063	−2.163	0.031	NA
ENSG00000262874	C19orf84	1.224	3.628	0.000	NA
ENSG00000233493	TMEM238	−1.064	−4.849	0.000	NA
ENSG00000179954	SSC5D	−1.192	−2.558	0.011	NA
ENSG00000196263	ZNF471	−1.153	−3.938	0.000	NA
ENSG00000211659	IGLV3-25	−1.369	−3.483	0.000	NA
ENSG00000264063	MIR3687-2	−1.068	−2.784	0.005	NA
ENSG00000215533	LINC00189	1.417	2.738	0.006	NA
ENSG00000236056	GAPDHP14	1.401	2.836	0.005	NA
ENSG00000255568	BRWD1-AS2	−1.119	−3.732	0.000	NA
ENSG00000210049	MT-TF	1.037	2.638	0.008	NA

Stat: statistics; padj: *p* value adjustable.

**Table 3:** Different expressed genes between HD gene carrier with depression and HD gene carrier without depression.

Gene ID	Gene Name	log2FoldChange	Stat	*p* Value	padj
ENSG00000130202	NECTIN2	−1.20871551	−2.27773	0.022743	0.999462
ENSG00000235169	SMIM1	1.286216213	2.0662	0.03881	0.999462
ENSG00000163646	CLRN1	−1.417542862	−2.08074	0.037458	0.999462
ENSG00000233058	LINC00884	−1.037747878	−2.89202	0.003828	0.999462
ENSG00000010030	ETV7	1.070004254	2.301148	0.021383	0.999462
ENSG00000215018	COL28A1	−1.217119591	−2.50457	0.01226	0.999462
ENSG00000175445	LPL	1.308483772	2.189831	0.028537	0.999462
ENSG00000178860	MSC	−1.100681038	−2.98191	0.002865	0.999462
ENSG00000159247	TUBBP5	−1.267966389	−2.35356	0.018595	0.999462
ENSG00000196565	HBG2	−1.350354678	−2.52451	0.011586	0.999462
ENSG00000251381	LINC00958	2.26201877	2.3048	0.021178	0.999462
ENSG00000254789	AC073172.1	−1.332994545	−2.7906	0.005261	0.999462
ENSG00000255508	AP002990.1	−1.058759746	−3.49951	0.000466	0.999462
ENSG00000078114	NEBL	2.623958542	3.216931	0.001296	0.999462
ENSG00000200830	RN7SKP134	−1.036440243	−2.74592	0.006034	0.999462
ENSG00000135116	HRK	−1.007746896	−2.79251	0.00523	0.999462
ENSG00000124107	SLPI	−1.250074721	−2.10266	0.035496	0.999462
ENSG00000226025	AC005515.1	1.025633489	2.438544	0.014747	0.999462
ENSG00000160233	LRRC3	−1.09935177	−2.93576	0.003327	0.999462

Stat: statistics; padj: *p* value adjustable.

**Table 4:** The 20 most significant different pathways between HD gene carriers and healthy control.

Genes	Process_Name	Significant_ Genes_Count	Total_Genes_ Group_Count	Percent_ Significant_Genes	*p*-Value	padj-Value
HBG2; HBA1;	GO:0015671~oxygen transport	2	14	14.286	0.00013	0.013186
ANK2; ABCC8;	GO:0043268~positive regulation of potassium ion transport	2	10	20.000	0.00007	0.013186
ANK2; SEMA3A;	GO:0002027~regulation of heart rate	2	31	6.452	0.00058	0.026314
FRMD6;	GO:0003383~apical constriction	1	3	33.333	0.00427	0.026314
ABCC8; KCNMA1;	GO:0006813~potassium ion transport	2	78	2.564	0.00337	0.026314
SSC5D; IGLV3-25; HBA1;	GO:0006898~receptor-mediated endocytosis	3	185	1.622	0.00110	0.026314
ALDH1L1;	GO:0009258~10-formyltetrahydrofolate catabolic process	1	2	50.000	0.00321	0.026314
ADGRG1;	GO:0010573~vascular endothelial growth factor production	1	3	33.333	0.00427	0.026314
ADGRG1;	GO:0021801~cerebral cortex radial glia guided migration	1	2	50.000	0.00321	0.026314
SEMA3A;	GO:0021828~gonadotrophin-releasing hormone neuronal migration to the hypothalamus	1	2	50.000	0.00321	0.026314
FRMD6;	GO:0032970~regulation of actin filament-based process	1	2	50.000	0.00321	0.026314
ANK2;	GO:0033292~T-tubule organization	1	3	33.333	0.00427	0.026314
KCNMA1;	GO:0034465~response to carbon monoxide	1	3	33.333	0.00427	0.026314
ANK2; FRMD6;	GO:0034613~cellular protein localization	2	40	5.000	0.00094	0.026314
ANK2;	GO:0036309~protein localization to M-band	1	2	50.000	0.00321	0.026314
ANK2;	GO:0036371~protein localization to T-tubule	1	1	100.000	0.00214	0.026314
SEMA3A;	GO:0036486~ventral trunk neural crest cell migration	1	3	33.333	0.00427	0.026314
SSC5D;	GO:0042494~detection of bacterial lipoprotein	1	1	100.000	0.00214	0.026314
SEMA3A;	GO:0048880~sensory system development	1	3	33.333	0.00427	0.026314
SSC5D; DEFA3;	GO:0050830~defense response to Gram-positive bacterium	2	66	3.030	0.00245	0.026314

padj-value: *p* value adjustable.

**Table 5:** The 20 most significant different pathways between HD gene carries with depression and HD gene carriers without depression.

Genes	Process_Name	Significant_ Genes_Count	Total_Genes_ Group_Count	Percent_ Significant_Genes	*p*-Value	padj-Value
NECTIN2;	GO:0002891~positive regulation of immunoglobulin mediated immune response	1	3	33.3333	0.00136	0.008475
SLPI; COL28A1;	GO:0010951~negative regulation of endopeptidase activity	2	124	1.6129	0.00084	0.008475
MSC;	GO:0014707~branchiomeric skeletal muscle development	1	3	33.3333	0.00136	0.008475
NECTIN2;	GO:0030382~sperm mitochondrion organization	1	2	50.0000	0.00102	0.008475
NECTIN2;	GO:0032990~cell part morphogenesis	1	1	100.0000	0.00068	0.008475
NECTIN2;	GO:0033005~positive regulation of mast cell activation	1	2	50.0000	0.00102	0.008475
LPL;	GO:0034371~chylomicron remodeling	1	3	33.3333	0.00136	0.008475
NECTIN2;	GO:0044406~adhesion of symbiont to host		3	33.3333	0.00136	0.008475
NECTIN2;	GO:0046814~coreceptor-mediated virion attachment to host cell	1	1	100.0000	0.00068	0.008475
NECTIN2;	GO:0051654~establishment of mitochondrion localization	1	2	50.0000	0.00102	0.008475
NECTIN2;	GO:0060370~susceptibility to T cell mediated cytotoxicity	1	3	33.3333	0.00136	0.008475
NEBL;	GO:0071691~cardiac muscle thin filament assembly	1	1	100.0000	0.00068	0.008475
NECTIN2;	GO:0042271~susceptibility to natural killer cell mediated cytotoxicity	1	4	25.0000	0.00169	0.009079
NECTIN2;	GO:0046596~regulation of viral entry into host cell	1	4	25.0000	0.00169	0.009079
NECTIN2;	GO:0002860~positive regulation of natural killer cell mediated cytotoxicity directed against tumor cell target	1	7	14.2857	0.00271	0.009770
NECTIN2;	GO:0007289~spermatid nucleus differentiation	1	8	12.5000	0.00305	0.009770
LPL;	GO:0010886~positive regulation of cholesterol storage	1	7	14.2857	0.00271	0.009770
LPL;	GO:0010890~positive regulation of sequestering of triglyceride	1	7	14.2857	0.00271	0.009770
NECTIN2;	GO:0019064~fusion of virus membrane with host plasma membrane	1	8	12.5000	0.00305	0.009770
HRK;	GO:0032464~positive regulation of protein homooligomerization	1	8	12.5000	0.00305	0.009770

padj-value: *p* value adjustable.
